# Glycemic control and its associated factors among diabetic heart failure outpatients at two major hospitals in Jordan

**DOI:** 10.1371/journal.pone.0285142

**Published:** 2023-10-05

**Authors:** Anan S. Jarab, Walid A. Al-Qerem, Hanan Hamam, Shrouq Abu Heshmeh, Sayer Al-Azzam, Tareq L. Mukattash, Eman A. Alefishat

**Affiliations:** 1 Department of Clinical Pharmacy, Faculty of Pharmacy, Jordan University of Science and Technology, Irbid, Jordan; 2 College of Pharmacy, Al Ain University, Abu Dhabi, United Arab Emirates; 3 Department of Pharmacy, Faculty of Pharmacy, Al-Zaytoonah University of Jordan, Amman, Jordan; 4 Department of Pharmacology, College of Medicine and Health Science, Khalifa University of Science and Technology, Abu Dhabi, United Arab Emirates; 5 Department Biopharmaceutics and Clinical Pharmacy, Faculty of Pharmacy, University of Jordan, Amman, Jordan; 6 Center for Biotechnology, Khalifa University of Science and Technology, Abu Dhabi, United Arab Emirates; HT Ong Heart Clinic, MALAYSIA

## Abstract

Patients with heart failure (HF) are generally at higher risk of developing type 2 diabetes and having uncontrolled blood glucose. Furthermore, the prevalence of uncontrolled blood glucose in patients with HF is largely unknown. Identifying the factors associated with poor blood glucose control is a preliminary step in the development of effective intervention programs. The current cross-sectional study was conducted at two major hospitals to explore the factors associated with blood glucose control among patients with heart failure and type 2 diabetes. In addition to sociodemographic, medical records were used to collect medical information and a validated questionnaire was used to evaluate medication adherence. Regression analysis showed that poor medication adherence (OR = 0.432; 95%CI 0.204–0.912; P<0.05) and increased white blood cells count (OR = 1.12; 95%CI 1.033–1.213; P<0.01) were associated with poor glycemic control. For enhancing blood glucose control among patients with HF and diabetes, future intervention programs should specifically target patients who have high WBC counts and poor medication.

## Introduction

Heart Failure (HF) is a cardiovascular disease (CVD) that results from cardiac abnormalities that lead to reduced cardiac output and/or elevated cardiac pressures at rest or during physical stress [1], therefore, negatively affecting the heart’s ability to pump enough blood to meet the body’s needs [2]. HF has been growing globally as a public health problem and one of the main causes of mortality and morbidity [3, 4]. HF affects about 64.3 million patients around the world [5]. CVDs, including HF, are responsible for about one-third of all global deaths, with more than three-quarters of mortalities occurring in developing countries [6]. Recent studies found that CVDs, including heart failure, contributed to about 37% of all deaths in Jordan [7]. Furthermore, HF results in frequent hospitalization and its management require approximately 1–2% of healthcare expenses [8–10].

Diabetes mellitus is a global public health concern and the seventh leading cause of death worldwide [11, 12]. It has been reported that the Middle East and North Africa (13) represent the second highest diabetes prevalence worldwide, with 54.8 million adults estimated to be living with diabetes [13, 14]. This number is expected to increase by 96% to reach 107.6 million by 2045 [14]. Previous studies reported that poor glycemic control was associated with an increased risk of visual impairment [15], kidney failure [16], and CVDs, including HF [17]. Recent evidence from the UK Prospective Diabetes Study (UKPDS), a clinical trial sample, suggests that poor glycemic control is associated with an increased risk of HF among patients with diabetes [18]. Diabetes is frequently observed in HF patients with a prevalence ranging from 10 to 30% and up to 40% in hospitalized subjects [11, 12, 19]. An earlier study was conducted to examine the association between glycemic control and the risk of HF in a population-based sample of adult patients with diabetes found that each 1% increase in HbA1c was associated with an 8% increased risk of HF, and an HbA1c ≥10, relative to HbA1c <7, was associated with 1.56-fold greater risk of HF [20]. Results also showed that the coexistence of diabetes and HF predicts a poor prognosis[20]. Previous observational studies [21, 22] and clinical trials [23–26] have demonstrated that diabetes is associated with increased mortality in HF patients. In the CHARM program of chronic HF, rates of HF hospitalization in patients with diabetes were also approximately twice the rates of those without diabetes [26].

Several factors have been identified to be associated with poor glycemic control in patients with diabetes in the literature. A study conducted in Ethiopia found that poor glycemic control was associated with illiteracy, and longer duration of diabetes [27]. Another study was conducted in the United States revealed that patients who were uninsured, had diabetes for a longer period of time, used insulin or multiple oral agents, or had high cholesterol levels had higher HbA1c values indicating poorer glycemic control [28]. Patients with HF are generally at higher risk of developing type 2 diabetes and having uncontrolled blood glucose. Furthermore, the prevalence of uncontrolled blood glucose in patients with HF is largely unknown. Uncontrolled blood glucose lead to progression of macrovascular complications in patients with existing cardiovascular disease, Nevertheless, limited research has been conducted to investigate the factors associated with poor glycemic control in patients with HF worldwide, and the current study is the first one to explore this in Jordan. Identifying the predictors of poor glycemic control is a preliminary step in the development of intervention programs which aim to improve glycemic control and hence health outcomes in patients with HF.

## Materials and methods

### Study design and settings

**Fig 1:**
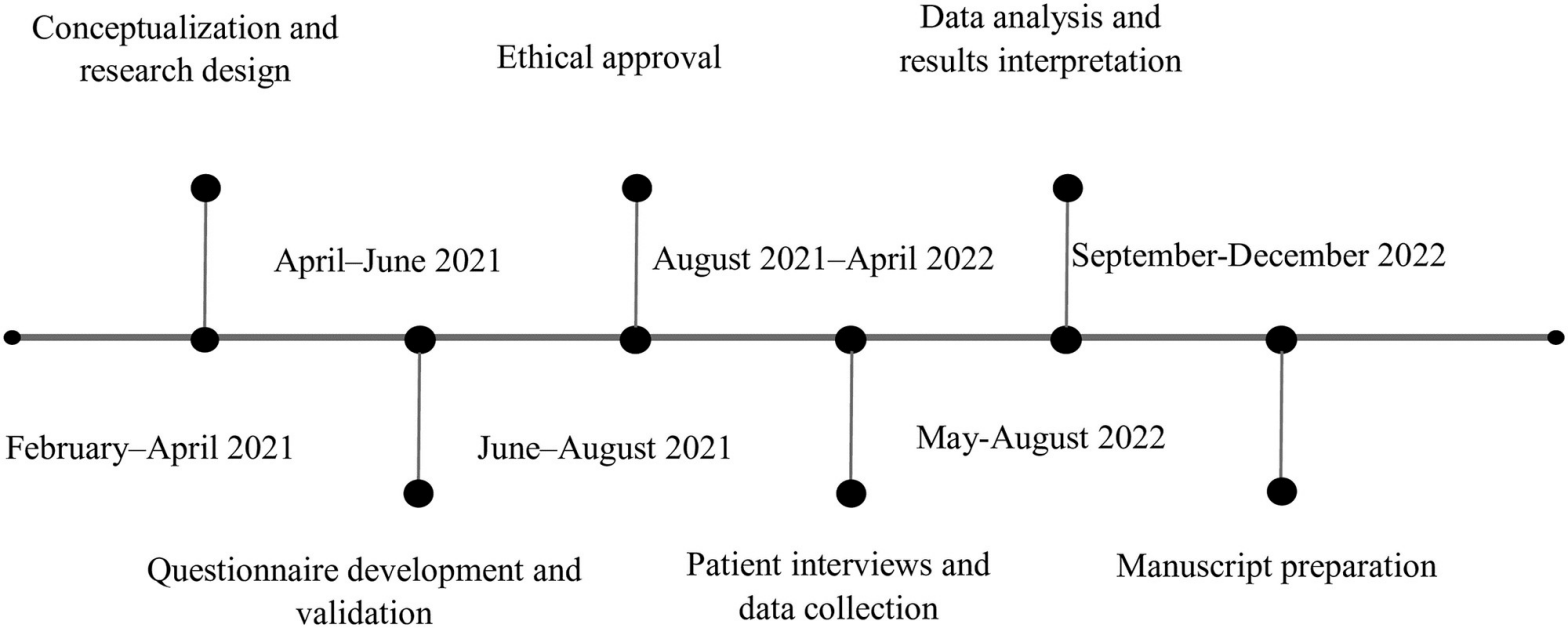
Study timeline.

The current cross-sectional study was conducted at the outpatient cardiology clinics in King Abdullah University Hospital and Al Bashir Hospital in the period from August 2021 through April 2022. The study timeline illustrates the research development and implementation steps throughout the study period (Fig 1).

### Sampling

Consecutive sampling technique was utilized in the present study. Patients who had HF and type 2 diabetes for at least 6 months, aged 18 years or older, had a cardiologist’s assignment of NYHA classification, were taking at least one HF medication, and agreed to participate in the study were invited to participate in the study. Patients with acute decompensation of HF or an active listing for heart transplantation and patients with cognitive impairment were excluded from the study. Eligible patients were informed that participation is voluntary, they have the right to withdraw at any time, and their medical care and treatment will not be affected whether they participate or not. They were also informed that the collected data would only be used for research purposes and would be saved at the Principal Investigator’s office to ensure confidentiality. Each interview took approximately 10–15 minutes to be completed. An informed consent form was obtained from each patient agreed to participate in the study.

### Ethical approval

The ethical approval was obtained from the Institutional Review Board (IRB) of KAUH at Jordan University of Science and Technology (Ref. # 32/141/2021).

### Data collection and study instruments

#### Sociodemographic data

The trained researcher HH used a custom-designed questionnaire and utilized the medical records to collect information about participants’ age, gender, body mass index (BMI), marital status, place of residency, living arrangements, education, occupation status, monthly income, smoking status, physical activity at an average of 30–45 minutes of walking per day on most days of the week, and family history of cardiovascular diseases.

#### Disease and medication-related data

The collected information also included NYHA heart failure classification, duration of HF, the presence and number of other comorbidities such as hypertension, diabetes, ischemic heart disease, chronic kidney disease, dyslipidemias and thyroid dysfunction. The researcher also collected information about the received medications, total number of medications, number of heart failure medications, frequency of taking medications, medications side effects, fears of medications side effects, medications satisfaction. The medical records were used to obtain biomedical data and laboratory tests including low-density lipoproteins (LDL), high-density lipoproteins (HDL), triglycerides, total cholesterol, hemoglobin HbA1c, random blood glucose, systolic blood pressure, diastolic blood pressure, ejection fraction, serum creatinine, white blood cells, red blood cells and hemoglobin (Hb). Based on the American Diabetes Association guidelines of HbA1c target in patients with diabetes and coexisting chronic disease, the patients were considered to have controlled blood glucose if HbA1c was < 8%. We defined uncontrolled diabetes as an HbA1c ≥ 8.0% in accordance with recommendations from the American Diabetes Association and the AHA/HFSA [29, 30].

#### The 4-item medication adherence scale

**Fig 2:**
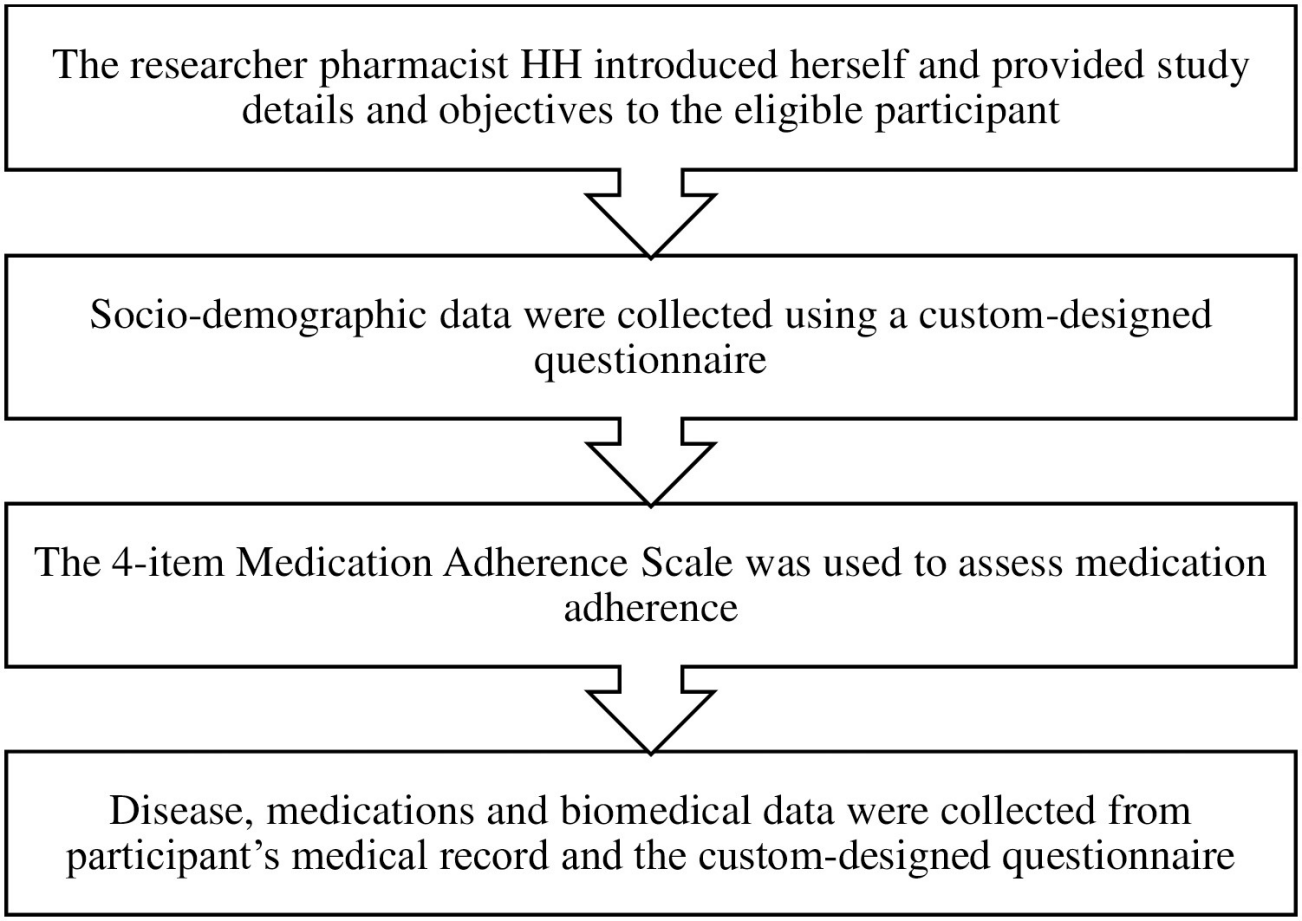
Interview chart.

This instrument was used to assess patients’ commitment to take their medications as prescribed; this simple, validated survey contains 4 questions, each question indicated a reason for medication omission such as forgetting, carelessness, or stopping when feeling better or worse [31]. One score was given for each ‘yes’ response, and each ‘no’ response was given a score of zero. The scores ranged from 0 to 4. According to this scale, adherence was divided into three groups: high for those scoring zero, medium for those scoring one or two, and low for those scoring three or four. The Arabic version has been used to assess medication adherence in patients with HF in the current study [32]. The surveys were completely self-reported, and patients who had difficulty completing questionnaires had the questionnaires read to them without giving any interpretation of the questions. The interview chart illustrates the steps of patient interviewing and the instruments utilized for this purpose (Fig 2).

### Data analysis

Using the Statistical Package for the Social Sciences (SPSS version 26 from IBM), descriptive and analytical statistics were conducted. Using the term of the mean (standard deviations), and frequencies (percentages) for descriptive analyses, continuous and categorical variables were described, respectively. The study sample’s differences according to glycemic control were tested using the independent sample t-test for normally distributed continuous variables and the Mann–Whitney U-test for non-normally distributed continuous variables. To figure out if there is an association between categorical variables and glycemic control, the Pearson Chi-square test was constructed, and the variables with a p-value < 0.2 were entered into the binary logistic regression to investigate the significant and independent predictors of glycemic control. Significance level was determined at P<0.05.

## Results

Out of 428 patients who were diagnosed with HF and type 2 diabetes, a total of 242 patients agreed to participate in the present study. The mean age was 64±10. The majority of the participants were males (59.1%), married (95%), living with their families (94.6%), living in the city (69.8%), did not complete their education (74%), unemployed or retired (81%), had a monthly income less than 500 JD (70.2%), non-smokers (73.1%), and were not physically active (88%). Table 1 represents the socio-demographic characteristics of the study participants.

**Table 1:** Demographic characteristics of the study participants (n = 242).

Characteristic	Mean (±SD^a^)	n (%)
**Age**	64 (±10)	
**Gender**		
Male		143 (59.1)
Female		99 (40.9)
**Body Mass Index (kg/m** ^**2**^ **)**		
Normal (≤24.9)		39 (16.7)
Overweight (25–29.9)		84 (36.1)
Obese (≥30)		110 (47.2)
**Marital status**		
Married		230 (95)
Single/ Divorced/ Widowed		12 (5)
**Living conditions**		
Living alone		13 (5.4)
Living with family/ other		229 (94.6)
**Residency**		
City		169 (69.8)
Countryside		73 (30.2)
**Education**		
University/ Collage		63 (26)
Less than collage		179 (74)
**Working status**		
Employed		46 (19)
Unemployed/ Retired		196 (81)
**Monthly income**		
Less than 500 JD ^b^		170 (70.2)
500–1000 JD ^b^		72 (29.8)
**Smoking status**		
Current Smoker		65 (26.9)
Non-smoker		177 (73.1)
**Performing regular physical activity** ^c^		
Yes		29 (12)
No		213 (88)
**Family history of CVD** ^d^		
Yes		112 (46.3)
No		130 (53.7)

^a^ Standard deviation;

^**b**^ JD: Jordanian Dinar;

^c^ 30–45 minutes of walking per day; on most days of the week,

^d^ cardiovascular diseases.

As shown in Tables 2 and 3, HF duration had a mean of 6.67 (± 5.75). The mean number of other comorbidities was 3 (±1). The most common comorbidities among those patients were hypertension (85.1%), and ischemic heart diseases (64%). The majority of patients were classified as group III/IV according to NYHA classification (76%). HF patients in our study were taking 9 (±3) medications on average, and the mean number of HF medications was 3 (±1). The most prescribed HF medications were beta-blockers (88.8%), loop diuretics (77.7%), and angiotensin converting enzyme inhibitors/angiotensin receptor blockers (69.4%). The majority of the patients were satisfied with their medications (74%), had no fears of medications’ side effects (69.8%), and did not experience any side effects (66.1%). The means of HbA1c, LDL, and Hb tests were 8.14 (±1.95), 2.34 (±0.94) mmol/l, and 12.63 (±2.16) g/dl, respectively.

**Table 2:** Medical characteristics of the study participants (n = 242).

Variables		n (%)	Mean (SD^a^)
**Duration of heart failure (years)**			6.67 (±5.75)
**Number of other chronic diseases**			3 (±1)
**NYHA** ^b^ **classification**	I/II	58 (24)	
	III/IV	184 (76)	
**Type of comorbidities**	Hypertension	206 (85.1)	
	Ischemic Heart Diseases	155 (64)	
	Dyslipidemia	82 (33.9)	
	Chronic Kidney Disease	43 (17.8)	
	Thyroid Dysfunction	22 (9.1)	
**Heart failure medications**	Aldosterone Antagonist	51 (21.1)	
	Digoxin	32 (13.2)	
	ACEIs ^c^/ARBs ^d^	168 (69.4)	
	Beta blocker	215 (88.8)	
	Loop diuretic	188 (77.7)	
	Vasodilator	44 (18.2)	
	Calcium Channel Blocker	58 (24)	
	Ivabradine	12 (5)	
	Valsartan/ Sacubitril	19 (7.9)	
**Other medications**	Anticoagulant	82 (33.9)	
	Statin	177 (73.1)	
**Number of Medications**			9 (±3)
**Number of heart failure medications**			3 (±1)
**Medications frequency**	Once	29 (12)	
	Twice	151 (62.4)	
	Thrice or more	62 (25.6)	
**Fear of medications’ side effects**	No	169 (69.8)	
	Yes	73 (30.2)	
**Medications satisfaction**	No	63 (26)	
	Yes	179 (74)	
**Experiencing side effects**	No	160 (66.1)	
	Yes	82 (33.9)	

^a^ Standard deviation;

^b^ The New York Heart Association Classification;

^**c**^ Angiotensin-converting enzyme inhibitors;

^d^ Angiotensin-receptor blockers.

**Table 3:** Biomedical variables of the study participants (n = 242).

Variable	Mean (±SD)
HbA1c (%)	8.14 (±1.95)
TGs (mmol/L)	2.076 (±1.27)
TC (mmol/L)	4.11 (±1.198)
LDL (mmol/L)	2.34 (±0.94)
HDL (mmol/L)	0.989 (±0.332)
Non-HDL Cholesterol (mmol/L)	2.91 (±1.38)
SCr (micmol/L)	116.11 (±81.91)
Hb (g/dL)	12.63 (±2.16)
WBCs count (*10^9^/L)	8.9 (±3.11)
RBCs count (*10^9^/L)	4.64 (±0.98)
EF (%)	42 (±10)
SBP (mmHg)	132 (±22)
DBP (mmHg)	77 (±12)

SD: Standard deviation; Hb: Hemoglobin; TGs: Triglycerides; TC: Total cholesterols; LDL: Low-density lipoproteins; HDL: High-density lipoproteins, SCr: Serum creatinine; WBCs: White blood cells; RBCs: Red blood cells; EF: Ejection fraction; SBP: Systolic blood pressure; DBP: Diastolic blood pressure.

According to the 4-item Medication Adherence Scale, the majority of the patients showed moderate adherence to HF medications (83.9%), 10.7% showed low adherence, while only 5.4% had high medication adherence. Results showed that 118 patients (48.8%) were considered to have controlled blood glucose (HbA1c < 8.0%) and 124 patients (51.2%) were considered to have uncontrolled blood glucose (HbA1c ≥ 8.0%). The variables that were significantly associated with glycemic control in the univariate analysis were age (P<0.01), gender (P<0.05), and WBC count (P<0.05). Binary logistic regression was conducted to explore the variables that were significantly and independently associated with glycemic control. Variables with P<0.2 in the univariate analysis including age, gender, smoking status, NYHA classification, thyroid dysfunction, ACEIs/ARBs use, MMAS-4, TGs level, HDL level, WBC count, and EF were included in the multivariate analysis model. Results revealed that patients who were moderately adherent had lower odds to be in the uncontrolled type 2 diabetes group when compared to patients who were poorly adherent (OR = 0.432; 95%CI 0.204–0.912; P = 0.028), and higher levels of WBC increased the odds to be in the uncontrolled type 2 diabetes group (OR = 1.12; 95%CI 1.033–1.213; P = 0.006). Table 4 demonstrates the multivariate analysis results of the variables associated with glycemic control in HF patients.

**Table 4:** Multivariate analysis of the variables associated with glycemic control.

Variable		OR (95% CI)	P
**White blood cells count**		1.12 (1.033–1.213)	0.006**
**Medication adherence**			
	Poor	Reference	0.028*
	Moderate	0.432 (0.204–0.912)	

*Significant at P<0.05,

** Significant at P<0.01.

## Discussion

Poor glycemic control has been associated with a negative impact on diabetic patients and the healthcare system, including increased healthcare costs, increased medication costs, and higher rates of hospitalization [33]. Around 10% to 47% of patients with HF were reported to have concomitant type 2 diabetes [34]. It is reported that type 2 diabetes is associated with two times increase in the risk of HF in males and a five times higher risk in females [35]. The coexistence of type 2 diabetes and HF is associated with poor prognosis in which mortality rates are about twice that of the non-diabetic population [26]. On the other hand, several studies examined the effect of glycemic control, and observed a positive effect on the primary prevention of HF. Every 1% reduction in hemoglobin HbA1c was found to be associated with a 16% reduction in the risk of the development of HF [18]. It is worth mentioning that treatment adjustment by healthcare providers in a timely manner has been reported to improve the control of diabetes and heart failure [18]. Several earlier studies were conducted to explore the variables associated with poor glycemic control among patients with type 2 diabetes; however, the current study is the first one implemented to investigate the factors associated with poor glycemic control among patients with HF.

The current study results found that nearly half of the patients who suffer from type 2 diabetes along with HF had poor glycemic control (48.8%), which was consistent with the findings reported in a US study that involved diabetic patients with a primary diagnosis of HF [36]. Another study reported an even higher percentage, with 74.2% of the participating patients showing inadequate glycemic control among patients with type 2 diabetes and HF in Malaysia [37]. The disappointing rates of poor glycemic control among diabetic patients with HF highlights the need for revealing the core influencing factors that hinder the achievement of blood sugar targets in these patients.

Our study results revealed that poor medication adherence was significantly associated with poor glycemic control. Consistent results were reported in a cohort study of over eleven thousand patients with type 2 diabetes who were followed from 1994 to 2006 and were found to have poor glycemic control due to medication non-adherence [38]. Another cohort study reported that poorly adherent patients with newly diagnosed diabetes had significantly higher HbA1c levels and higher hospitalization or emergency department visits over a 2-year follow up period when compared with those who were fully adherent [39]. Poor medication adherence was also linked to higher HbA1c levels, and poor glycemic control in diabetic patients who participated in an Egyptian study [40]. Furthermore, glycemic control was found to be significantly affected by medication non-adherence among patients with type 2 diabetes who participated in several other studies conducted in Palestine [41], USA [42], China [43], and India [44]. Therefore, healthcare professionals should pay close attention to this problematic issue and exert efforts to solve it by implementing advanced strategies that aim at improving medication adherence through overcoming barriers to optimal medication adherence, and thus enhancing glycemic control in patients with type 2 diabetes, with special attention to those with additional comorbidities.

Another significant predictor of poor glycemic control in the present study was the presence of a higher white blood cell count (WBC). Elevated WBC count may reflect chronic low-grade inflammation that involves the activation of the immune system and the release of inflammatory cytokines, which was thought to play a role in insulin resistance and type 2 diabetes pathogenesis [45, 46]. An earlier study reported an association between high WBC count and worsening of insulin action and the development of type 2 diabetes in the participating individuals, supporting the hypothesis that a chronic activation of the immune system may have a role in the pathogenesis of type 2 diabetes [47], as the reduction of insulin sensitivity aid in poor glycemic control. Another study which was conducted among high-fat diet fed mice reported that treatment of hepatocytes with neutrophil elastase, which is a protease secreted by neutrophils during inflammation, caused cellular insulin resistance and that omission of this protease in obese mice was associated with decreased tissue inflammation, which was also accompanied by improved glucose tolerance and increased insulin sensitivity [48]. These findings shed light on the need for addressing the causes of increased WBC count in patients with type 2 diabetes and HF in order to prevent a chronic inflammation state, subsequently, improving glycemic control in these patients. However, the use of C-reactive protein would lead to more specific finding when compared with WBCs count.

The current study has some limitations, since it is a cross-sectional study, cause-effect relationship cannot be established. Furthermore, due to social desirability bias, the self-report method used in the current survey may have affected the accuracy the data. Despite these limitations, this study provided unique insights into the predictors of poor glycemic control among Jordanian patients with HF and type 2 diabetes.

## Conclusions

Glycemic control in patients with type 2 diabetes and HF was inadequate in this study. Medication non-adherence and high WBC count were found to be associated with poor glycemic control. Future intervention programs should emphasize the importance of medication adherence in controlling blood sugar, particularly in patients with elevated WBCs count. Further prospective intervention studies need to address whether good glycemic control and reducing inflammation would have a positive impact on patients with HF and type 2 diabetes.
